# High-yield overproduction and purification of human aquaporins from *Pichia pastoris*

**DOI:** 10.1016/j.xpro.2022.101298

**Published:** 2022-04-12

**Authors:** Tamim Al-Jubair, Jonas Hyld Steffen, Julie Winkel Missel, Philip Kitchen, Mootaz M. Salman, Roslyn M. Bill, Pontus Gourdon, Susanna Törnroth-Horsefield

**Affiliations:** 1 Department of Biochemistry and Structural Biology, Lund University, P.O. Box 124, 221 00 Lund, Sweden; 2 Department of Biomedical Sciences, University of Copenhagen, 2200 Copenhagen, Denmark; 3 College of Health and Life Sciences, Aston University, Aston Triangle, B4 7ET Birmingham, UK; 4 Department of Physiology, Anatomy and Genetics, Kavli Institute for NanoScience Discovery, University of Oxford, Parks Road, OX1 3PT Oxford, UK; 5 Oxford Parkinson’s Disease Centre, University of Oxford, Oxford, UK; 6 Department of Experimental Medical Science, Lund University, P.O. Box 118, 221 00 Lund, Sweden

**Keywords:** Cell Membrane, Molecular Biology, Protein Biochemistry, Protein expression and purification

## Abstract

Aquaporins (AQPs) are membrane-bound water channels that play crucial roles in maintaining the water homeostasis of the human body. Here, we present a protocol for high-yield recombinant expression of human AQPs in the methylotropic yeast *Pichia pastoris* and subsequent AQP purification. The protocol typically yields 1–5 mg AQP per g of yeast cell at >95% purity and is compatible with any membrane protein cloned into *Pichia pastoris*, although expression levels may vary.

For complete details on the use and execution of this protocol, please refer to Kitchen et al. (2020) and Frick et al. (2014).

## Highlights

• Human AQPs can be overproduced at high yields in *Pichia pastoris*
• Our purification protocol leads to highly pure and homogenous protein sample
• The protocol is suitable for any human AQP as well as other membrane proteins

## Graphical abstract

**Figure:**
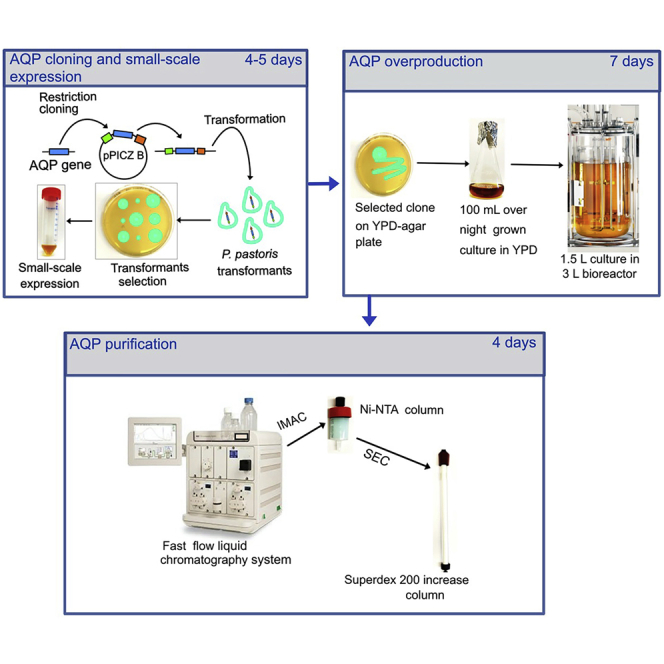

## Before you begin

We have successfully expressed several human AQPs as well as other eukaryotic membrane proteins in *Pichia pastoris* using the pPICZ expression vectors and the X-33 *Pichia* strain from Invitrogen^TM^ (Thermo Fisher Scientific, US). In this system, the gene of interest is integrated into the *Pichia* genome and expressed under the control of the *AOX1*-promotor.

### Prepare plasmid and transform it into *Pichia pastoris*

**Timing: 1+ days**

1. Design AQP gene constructs for overproduction in *Pichia pastoris.* The construct should include the following:
  a. a codon optimized gene for expressing the AQP of interest in yeast.
  b. a poly-histidine tag (6–10 histidine residues).
  c. a protease cleavage site for tag removal. We typically use a TEV-site with the sequence ENLYFQG.

***Optional:*** It is often advantageous to have a linker between the protease site and the protein open reading frame. When using a TEV-site we use a 7-amino acid linker with the sequence DYDIPTT.

***Note:*** Since the position of the tag can have a substantial effect on the expression levels, we strongly recommend to make both N- and C-terminally tagged constructs.

2. Sub-clone the AQP gene into one of the pPICZ-vectors from Invitrogen^TM^.
3. Transform the plasmid into the X-33 *Pichia* strain by electroporation as described in the EasySelect Pichia Expression Kit manual (see key resources table).

## Key resources table

**Table:** 

REAGENT or RESOURCE	SOURCE	IDENTIFIER
**Antibodies**		

Goat Anti-Mouse IgG Peroxidase-Labeled Antibody (Secondary antibody, 1:5,000 dilution)	SeraCare	Cat# 5220-0341; RRID: AB_2891080
Mouse Anti 6× His Monoclonal Antibody (Primary antibody, 1:5,000 dilution)	Takara Bio	Cat# 631212

**Chemicals, peptides, and recombinant proteins**		

Ammonia 25%	Merck	Cat# 1054325000
Boric Acid	Sigma-Aldrich	Cat# B6768-500G
Bromophenol Blue	Sigma-Aldrich	Cat# B0126-25G
CoCl_2_	Sigma-Aldrich	Cat# 449776-5G
CaSO_4_.2H_2_O	Merck	Cat# C3771-500G
CuSO_4_	Sigma-Aldrich	Cat# 451657-10G
D-Biotin	Thermo Fisher Scientific	Cat# B1595
Dextrose	Sigma-Aldrich	Cat# G8270-5KG
DTT (dithiothreitol)	Thermo Fisher Scientific	Cat# R0862
EDTA	Thermo Fisher Scientific	Cat# 15576028
Ethanol 70%	Solveco	Art Nr# 1047, 1022
FeSO_4._7H_2_O	Sigma-Aldrich	Cat# F7002-250G
Glycerol100%	Sigma-Aldrich	Cat# G5516-1L
HCL 37%	Sigma-Aldrich	Cat# 320331-500ML
H_3_PO_4_ 85%	Sigma-Aldrich	Cat# 695017-2.5L
H_2_SO_4_	Merck	Cat# 84727-500ML
Imidazole	Sigma-Aldrich	Cat# 2399-500G
Isopropanol 100%	Sigma-Aldrich	cat# I9516-1L
K_2_SO_4_	Sigma-Aldrich	Cat# P0772-250G
KH2Po4 (Potassium dihydrogen phosphate)	Merck	Cat# 1051080500
K2HPo4 (Potassium phosphate dibasic)	Merck	Cat# 60353-1KG
KOH	Merck	Cat# 1050330500
MgSO_4_. 7H_2_O	Sigma-Aldrich	Cat# 63138-250G
MnSO_4._ H_2_O	Sigma-Aldrich	Cat# M7634-500G
Methanol 99%	Thermo Fisher Scientific	Cat# ALF-L13255-AU
β-(N-Morpholino)ethanesulphonic acid (MES), anhydrous ≥99%	VWR	Cat# E183-500G
NaCl	Merck	Cat# S9888-5KG
NaI	Sigma-Aldrich	Cat# 409286-10G
Na_2_MoO_4_	Sigma-Aldrich	Cat# 243655-100G
Needle	Fisher Scientific	Cat# 14-840-88
n-Decyl-β-D-Maltopyranoside, Anagrade (DDM), Anagrade	Anatrace	Cat# D322LA 25 GM
n-Decyl-β-D-Maltopyranoside (DM), Anagrade	Anatrace	Cat# D322 25 GM
n-Octyl Glucoside, Anagrade	Anatrace	Cat# O311HA 25 GM
Nonyl β-D-glucopyranoside Anagrade	Anatrace	Cat# M324 25 GM
N-Tetradecyl-N-N-Dimethyldodecylamine N-oxide (LDAO), Anagrade	Anatrace	Cat# T360 25 GM
NuPAGE 4–12% Bis-Tris gel	Invitrogen	Cat# NP0329BOX
Octyl Glucose Neopentyl Glycol (OGNG)	Anatrace	Cat# NG311 25 GM
Peptone	Duchefa Biochemie	Cat# P1328.1000
Phenylmethylsulfonyl fluoride (PMSF)	Thermo Fisher Scientific	Cat# 36978
Polypropylene glycol P 2,000	Sigma-Aldrich	Cat# 81380-1L
Sodium Dodecyl Sulfate (SDS)	Thermo Fisher Scientific	Cat# 15525017
SimplyBlue SafeStain	Invitrogen	Cat# LC6065
Spectra Multicolor Broad Range Protein Ladder	Thermo Fisher Scientific	Cat# 26634
Tween™ 20	Thermo Fisher Scientific	Cat# 28321
Tris base	Thermo Fisher Scientific	Cat# 17926
Urea	Invitrogen	Cat# AM9902
Yeast Extract	Sigma-Aldrich	Cat# Y1000-500G
YPD-agar	In this paper	N/A
YPD-medium	In this paper	N/A
Yeast Nitrogen Base Without Amino Acids and Ammonium Sulfate (YNB)	Merck	Cat# Y1251-100G
ZnCl_2_	Sigma-Aldrich	Cat# 429430-25G
Zeocin	Thermo Fisher Scientific	Cat# R25001

**Critical commercial assays**		

ECL^TM^ Prime Western Blotting Detection Reagent	Merck	Cat# GERPN2236

**Experimental models: Organisms/strains**		

P. pastoris X-33	Included in EasySelect *Pichia* Expression Kit by Thermo Fisher	Cat# V190-20

**Recombinant DNA**		

*AQP* gene with His Tag and TEV cleavage site	Synthesis by GenScript	N/A
pPICZ-vector	Included in EasySelect *Pichia* Expression Kit by Thermo Fisher	Cat# V190-20

**Software and algorithms**		

GraphPad Prism Version 9	GraphPad Prism Software	https://www.graphpad.com/support/faq/prism-900-release-notes/
Phantom Software	Phantom Technologies	https://www.phantom-project.org/software

**Other**		

0.2 μm syringe filters	Merck	Cat# CLS431219
0.5 mm diameter Glass Beads	Techtum	Cat# 11079105
1.5 mL tube	Fisher Scientific	Cat# 50-809-150
3 L Fermentor	Belach Bioteknik AB	N/A
10 mL Potter-Elvehjem Tissue Grinder	DWK Life Sciences	Cat# 358039
15 mL Centrifuge Tubes	SARSTEDT AG & Co. KG	Cat# 62554502
1 L polypropylene bottle	Beckman Coulter	Cat# A98813
26.3 mL, Polycarbonate Bottle	Beckman Coulter	Cat# 355654
45 Ti Fixed-Angle Titanium Rotor	Beckman Coulter	Cat# 339160
50 mL Falcon Tube	SARSTEDT AG & Co. KG	Art Nr# WW-866864
50 mL Super Loop	Cytiva	Cat# 19785001
70 mL, Polycarbonate Bottle	Beckman Coulter	Pro Nr# 355655
70 Ti Fixed-Angle Titanium Rotor	Beckman Coulter	Pro Nr# 337922
96 Deep Well plate	Merck	Cat# P8116-50EA
400 mL glass beaker	Sigma-Aldrich	Cat# Z740577
500 mL Polypropylene screw cap centrifuge bottle	Thermo Fisher Scientific	Pro code# 10343871
Nitrocellulose Membrane (0.45 μM)	Cytiva	Cat# CYTIVA 10600007
Centrifuge	Beckman Coulter	Cat# Avanti® J-26 XP
Analytical Ultracentrifuge	Beckman Coulter	Cat# Optima XL-I
Bead Beater HAMILTON BEACH 908 BASE	BioSpec Products	Model# 1107900
Stainless Steel Chamber Jar, 350 mL	BioSpec Products	Cat# 60801
Gel Imaging System	Syngene	Model# PXi/PXi Touch Series
HisTrap HP Pre-packed 5 mL Columns	Cytiva	Pro Code# 11773209
JLA-10.500 rotor	Beckman Coulter	Cat# JLA-10.500 m.V.
NGC chromatography system	Bio-Rad	Model# Quest 10 Plus #7880003
Protein Concentrator, 30 KDa	Thermo Fisher Scientific	Cat# 88531
Superdex 200 increase 10/300 GL column	Cytiva	Cat# 17-5175-01
XCell *SureLock* Mini-Cell	Invitrogen	Cat# EI0001
XCell SureLock Power Supplier	Invitrogen	Cat# EI8600
XCell II^TM^ Blot Module	Invitrogen	Cat# EI9051

## Materials and equipment

***Alternatives:*** In the key resources table, we have listed the instrument makes and models and also the chemicals used in our laboratory. However, these specific models and manufacturers are not crucial for the success of the protocol. There are also alternative *Pichia* strains and plasmids that can be used, if desired.

**Table:** Yeast Extract Peptone Dextrose (YPD)-medium/YPD-agar (Dissolve yeast extract and peptone in 900 mL water. Add agar for making YPD plates before autoclaving. Once the autoclaved medium has cooled down to room temperature, add sterile-filtered dextrose Liquid medium)

Reagent	YPD-medium		YPD-agar	
	Final concentration	Amount	Final concentration	Amount
Yeast extract	1% (w/v)	10 g	1% (w/v)	10 g
Peptone	2% (w/v)	20 g	2% (w/v)	20 g
Agar			2% (w/v)	20 g
De-ionized H_2_O		up to 900 mL		up to 900 mL
20% Dextrose (w/v) stock	2% (v/v)	100 mL	2% (v/v)	100 mL
**Total**		**1,000 mL**		**1,000 mL**

Store YPD liquid medium at 22°C and YPD plates at 4°C for a maximum of 3 months.

**Table:** BMGY/BMMY Medium (Dissolve 10 g yeast extract, 20 g peptone in 700 mL water first, autoclave and cool down to room temperature then mix sterile filtered potassium phosphate buffer, YNB, biotin and 100 mL of 10% (v/v) glycerol or 5% (v/v) methanol)

Reagent	Final concentration	Amount
Yeast extract	1% (w/v)	10 g
Peptone	2% (w/v)	20 g
De-ionized H_2_O		700 mL
1 M Potassium phosphate pH 6.0 stock	100 mM	100 mL
10× YNB stock	1.34% (v/v)	100 mL
10% (v/v) Glycerol (BMGY) or 5% (v/v) Methanol (BMMY)	glycerol 1% (v/v) or 0.5% (v/v) Methanol	100 mL
500× Biotin	4 × 10^-5^% (v/v)	2 mL
**Total**		**1,000 mL**

Store media at 4°C for a maximum of 2 months.

**Table:** 2× SDS-PAGE loading-buffer

Reagent	Final concentration	Amount
Tris (1 M, pH 6.8)	100 mM	1 mL
20% (w/v) SDS stock	4% (v/v) SDS	2 mL
80% (v/v) glycerol stock	20% (v/v) glycerol	2.5 mL
1 M DTT stock	200 mM DTT	2 mL
1% (v/v) bromophenol blue stock	0.04% (v/v) bromophenol blue	0.4 mL
De-ionized H_2_O		2.1 mL
**Total**		**10 mL**

Aliquot in 1 mL and store at −20°C for a maximum of 1 year.

**Table:** SDS running-buffer

Reagent	Final concentration	Amount
MES anhydrous	50 mM	9.76 g
Tris base	50 mM	6.06 g
SDS	1% (w/v)	1 g
EDTA	1 mM	0.3
De-ionized H_2_O		up to 1,000 mL
**Total**		**1,000 mL**

Store the running buffer at 22°C and use maximum 3 times.

**Table:** Tris-buffered saline with Tween 20 (TBST) (Prepare fresh)

Reagent	Final concentration	Amount
1 M Tris pH 7.6	20 mM	10 mL
5 M NaCl	150 mM	15 mL
Tween 20	0.2% (v/v)	1 mL
De-ionized H_2_O		up to 500 mL
**Total**		**500 mL**

Store at 4°C for a maximum of 3 months.

**Table:** Basal Salt Medium (BSM)

Reagent	Final concentration	Amount
Calcium sulphate	0.093% (w/v)	1.395 g
Potassium sulphate	1.82% (w/v)	27.3 g
Magnesium sulphate 7× H_2_O	1.49% (w/v)	22.35 g
Potassium hydroxide	0.41% (w/v)	6.195 g
Glycerol 100%	4.02% (w/v)	60.3 mL
85% Phosphoric acid	2.67% (v/v)	40.05 mL
De-ionized H_2_O		up to 1,500 mL
**Total**		**1,500 mL**

Prepare fresh.

**Table:** *Pichia* Trace Metals (PTM)

Reagent	Final concentration	Amount
Cupric sulphate	0.6% (w/v)	0.6 g
Sodium iodide	0.008% (w/v)	0.008 g
Manganese sulphate × H_2_O	0.3% (w/v)	0.3 g
Sodium molybdate ×2 H_2_O	0.02% (w/v)	0.02 g
Boric acid	0.002% (w/v)	0.002 g
Cobalt chloride	0.05% (w/v)	0.05 g
Zink chloride	2% (w/v)	2 g
Ferrous sulphate ×7 H_2_O	6.5% (w/v)	6.5 g
Biotin	0.02% (w/v)	0.02 g
Sulphuric acid	0.5% (w/v)	500 μL
De-ionized H_2_O		up to 100 mL
**Total**		**500 mL**

Filter sterile, aliquot in 20 mL and store at 22°C for a maximum of 6 months.

**Table:** Anti-foam solution

Reagent	Final concentration	Amount
Polypropylene glycol P 2,000	50% (v/v)	10 mL
De-ionized H_2_O		10 mL
**Total**		**20 mL**

Autoclave and store at 22°C for a maximum of 6 months.

**Table:** Breaking-buffer (Adjust the pH to 7.5)

Reagent	Final concentration	Amount
KH_2_PO_4_	0.22% (w/v)	2.2727 g
K_2_HPO_4_	0.58% (w/v)	5.8002 g
Glycerol 100%	5% (v/v)	5 mL
De-ionized H_2_O		up to 1,000 mL
**Total**		**1,000 mL**

Store at 4°C for a maximum of 2 months.

**Table:** Protease inhibitor, 100 mM phenylmethylsulfonyl fluoride (PMSF) solution

Reagent	Final concentration	Amount
phenylmethylsulfonyl fluoride	100 mM	0.35 g
100% Isopropanol		up to 20 mL
**Total**		**20 mL**

Aliquot in 2 mL and store at −20°C for a maximum of 6 months.

**Table:** Urea-buffer

Reagent	Final concentration	Amount
1 M tris pH 9.5 stock	5 mM	5 mL
Urea	4 M urea	240.24 g
0.5 M EDTA stock, pH 9	2 mM EDTA	4 mL
De-ionized H_2_O		up to 1,000 mL
**Total**		**1,000 mL**

Store at 4°C for a maximum of 3 months.

**Table:** Membrane-buffer

Reagent	Final concentration	Amount
1 M Tris pH 8.0	20 mM	20 mL
5 M NaCl	20 mM	4 mL
50% (v/v) glycerol stock	10% (v/v)	200 mL
De-ionized H_2_O		up to 1,000 mL
**Total**		**1,000 mL**

Store at 4°C for a maximum of 2 months.

**Table:** Solubilization-buffer

Reagent	Final concentration	Amount
1 M Tris pH 8.0	20 mM	10 mL
5 M NaCl	300 mM	30 mL
De-ionized H_2_O		up to 500 mL
**Total**		**500 mL**

Store at 4°C for a maximum of 2 months.

**Table:** Buffer -A, -B and -C (Adjust the pH to 8.0 after dissolving the imidazole)

Reagent	Buffer-A (10 mM imidazole)		Buffer-B (75 mM imidazole)		Buffer-C (300 mM imidazole)	
	Final concentration	Amount	Final concentration	Amount	Final concentration	Amount
1 M Tris pH 8.0 stock	20 mM	1 mL	20 mM	1 mL	20 mM	1 mL
Imidazole	10 mM	0. 03 g	75 mM	0.25 g	300 mM	1.02 g
OG	1% (w/v)	0.5 g	1% (w/v)	0.5 g	1% (w/v)	0.5 g
5 M NaCl Stock	300 mM	3 mL	300 mM	3 mL	300 mM	3 mL
De-ionized H_2_O		up to 50 mL		up to 50 mL		up to 50 mL
Total		50 mL		50 mL		50 mL

Store buffer with the added detergent at 4°C for a maximum of 3 days.

**Table:** SEC-buffer

Reagent	SEC-buffer	
	Final concentration	Amount
1 M Tris pH 8.0 stock	20 mM	4 mL
OG	1% (w/v)	2 g
5 M NaCl Stock	300 mM	12 mL
De-ionized H_2_O		up to 200 mL
Total		200 mL

Store buffer with the added detergent at 4°C for a maximum of 3 days.

## Step-by-step method details

### Select clones with higher AQP gene copy number

**Timing: 2 days**

Overproduction of the desired protein often depends on the copy number of the respective gene incorporated in the host genome. For *Pichia*, this sometimes leads to so-called jackpot clones with substantially higher expression levels than the other clones. To evaluate the expression level, we follow a somewhat modified protocol to that described in the EasySelect Pichia Expression Kit manual for screening AQP expression levels in *Pichia*, see below.

1. Draw a numbered grid (1–60) on the petri dish of an YPD-agar plate containing 2,000 μg/mL zeocin.
2. Randomly select 60 colonies from the transformation plate and spot them on the high-zeocin (2,000 μg/mL) using sterile 10 μL pipet tips.

**CRITICAL:** It is important to select colonies spread out over the whole transformation plate. If the transformation plate is older than 1 week, the colonies should first be spotted on an YPD-agar plate with 100 μg/mL zeocin and grown for 2 days at 30°C before being transferred to the high-zeocin plate (2,000 μg/mL).

**Figure 1:**
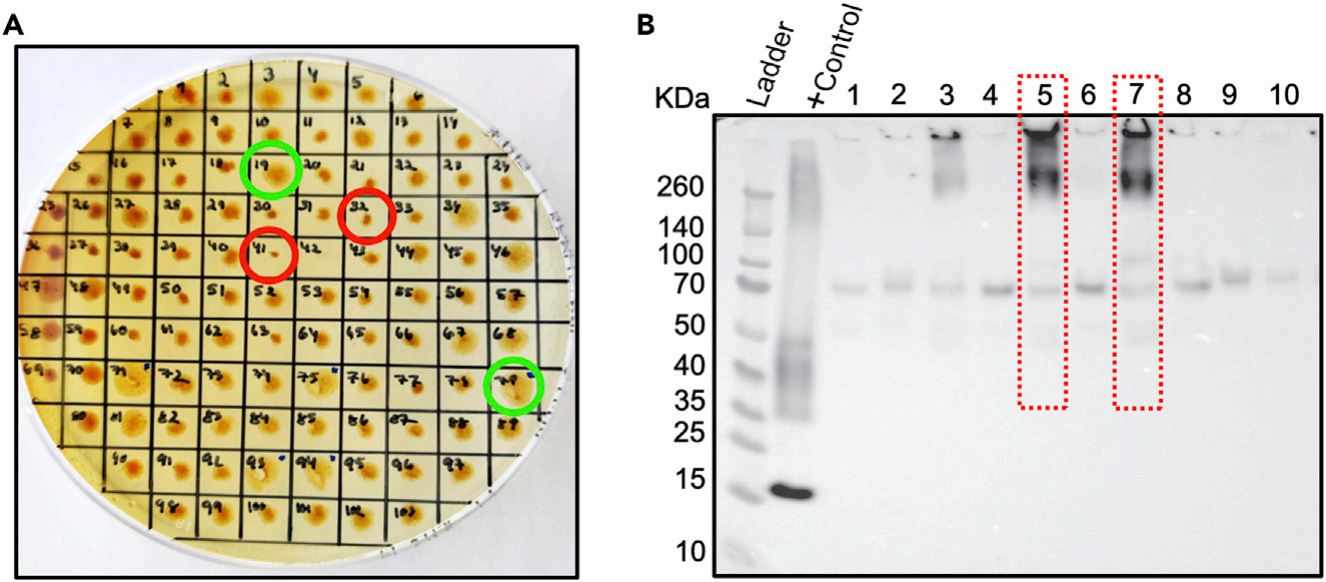
Small-scale screening of AQP expression in *Pichia pastoris* (A and B) (A) *P. pastoris* transformants grown on a YPD-agar plate containing 2,000 μg/mL zeocin. The red and green circles indicate colonies that are expected to have lower (red) and higher (green) copy number of the AQP gene, as estimated by their size. (B) Western blot result from small-scale expression analysis. Clones corresponding to lane 5 and 7 have comparatively higher band intensity (marked with red dotted boxes), demonstrating higher expression of the target protein.

***Note:*** A larger colony size on the high zeocin plate correlates with higher resistance to zeocin due to multiple insertion events into the genome and therefore also with higher expression levels of the protein of interest (Öberg et al., 2011).

3. Incubate the plate at 30°C for 2 days.
4. Select a minimum of 10 colonies that look larger in size than the others for small-scale expression analysis (Figure 1A).

### Grow selected colonies at small scale

**Timing: 4 days**

5. Grow each selected colony separately in 5 mL buffered complex glycerol (BMGY) medium in a 50 mL Falcon tube overnight with 200–300 rpm rotation at 30°C. Keep the lids loosely attached to the tubes to allow for proper aeration.
6. Check the optical density of the culture at 600 nm (OD600). Transfer the volume of cells that is equivalent to an OD600 of 1 in 5 mL to a new 50 mL Falcon tube. Add complex methanol (BMMY) medium to a final volume of 5 mL.

***Note:*** Methanol replaces glycerol as the carbon source and will induce the expression of the AQP gene.

7. For each clone, prepare 0 h induction samples for a western blot as follows:
  a. Transfer 1 mL of the BMMY-sample (1 OD-unit) into a 1.5 mL tube.
  b. Centrifuge at 15,000 × *g* for 5 min.
  c. Remove the supernatant and freeze the cell pellet for later use as the 0 h induced sample in the western blot.
8. Grow the remaining 4 mL of the BMMY-culture for 24 h with 200–300 rpm rotation at 30°C.
9. Check the OD_600_ of each culture and prepare 24 h induction samples for the western blot as in step 7.
10. Add 100% methanol to rest of the culture to a final concentration of 0.5% (v/v) methanol and grow for another 24 h.

**CRITICAL:** Be careful not to add more methanol than needed. It is toxic for the cells and accumulates if present in higher amounts than needed to sustain growth.

11. Check the OD_600_ at 48 h and prepare 48 h induction samples for the Western blot as described in step 7.

**Pause point:** Cells can be stored at −20°C for a month and at −80°C for years before analyzing the samples by the western blot method.

***Optional:*** If desired, the cultures can be grown for longer and samples taken out at later time points in order to determine the optimal induction time before harvesting. In our experience, 48 h is sufficient for most AQPs, however, 72 h have been shown to be beneficial in some cases. Please note that, since the methanol is consumed, it is essential to add methanol to a final concentration of 0.5% (v/v) every 24 h.

### Analyze AQP expression levels by western blot

12. Thaw the frozen samples that were collected at different induction time points.
13. Resuspend the cell pellet in 40 μL of 2× SDS loading-buffer and heat the samples for 5 min at 95°C.

**CRITICAL:** Do not centrifuge the samples after heating as this may pellet down the membrane fraction, resulting in a false negative result in the small-scale expression analysis. Also, do not vortex the sample in order to resuspend the pellet as this may result in foaming. Instead, gently pipette up and down 4–5 times before loading on SDS-PAGE.

***Optional:*** If desired, a very small membrane fraction of the samples can be isolated for small-scale expression analysis. To do this, resuspend the thawed cell pellet in 100 μL of breaking-buffer and place on ice. Add an equal volume of acid-washed glass beads. Vortex 8 × 30 s with 30 s incubation on ice in between. Centrifuge at 10,000 × *g* for 10 min in a desktop centrifuge at 4°C. Transfer the supernatant to a fresh microcentrifuge tube and pellet down the crude membrane by centrifuging at 20,000 × *g* at 4°C for 1 h. Resuspend crude membranes in 30 μL of 2× SDS-PAGE loading-buffer. Alternatively, the crude membranes can be pelleted down using a desktop ultracentrifuge (120,000 × *g*, for 1 h at 4°C), however, we find the recovery from centrifugation in a desktop centrifuge is sufficient for this analysis, and as the final pellet is less compact, it is easier to resuspend in SDS-PAGE loading-buffer. Load 10 μL for SDS-PAGE and follow the same protocol as for the whole cell fraction.

14. Run the samples on an SDS-PAGE gel. We use NuPAGE 4%–12% Bis-Tris gels (Thermo Fisher Scientific) in an Xcell SureLock Mini-Cell chamber (see key resources table).
  a. Assemble the chamber with the gel according to the manufacturer’s instructions.
  b. Fill up the chamber with SDS running-buffer.
  c. Load 10 μL of each sample in each well of the gel, leaving one well empty for the protein ladder.
  d. Load 5 μL pre-stained protein ladder (Spectra Multicolor Broad Range Protein Ladder, Thermo Fisher) in the empty well.
  e. Run the gel at 200 volts (constant) for 35 min (see key resources table).
15. Transfer the protein bands from the gel to a nitrocellulose (Amersham Protran 0.45 μM NC) or polyvinylidene diflouride (PVDF) blotting membrane. We use an XCell II Blot Module and follow the manufacturer’s instructions.
16. Block the membrane using 5% (w/v) dry milk in tris-buffered saline with 0.2% (v/v) Tween 20 (TBST) for 1 h at 22°C.
17. Incubate with a primary anti-His monoclonal antibody (5,000× dilution in TBST with 5% (w/v) dry milk) for 1 h at 22°C.
18. Wash the membrane for 3 × 10 min in TBST.
19. Incubate the membrane with a suitable horseradish peroxidase (HRP)-conjugated secondary antibody (5,000× dilution in TBST with 5% (w/v) dry milk) for 1 h at 22°C.
20. Wash the membrane for 3 × 10 min in TBST.
21. Develop the membrane with ECL Prime Western Blotting Detection Reagent (see key resources table) following the manufacturer’s protocol.
22. Analyze the chemiluminescence using a western blot imaging system (see key resources table).
23. Compare the intensity of the western blot signal in the different clones in order to identify the one with the highest AQP expression level (Figure 1B).

***Note:*** Since AQPs are integral membrane proteins, they often do not migrate according to the molecular weight and, also for purified protein, we most often observe bands for multiple oligomeric states, including those larger than the physiologically relevant tetramer. When estimating the expression level, it is important to take the intensity of all these bands into consideration. When using cellular lysates as samples for western blot, we frequently observe high molecular weight oligomers, as seen for AQP4 (Figure 1B). Nevertheless, the band intensity still correlates to the final protein yield for the specific clone and the protein is stable and homogenous after purification (Kitchen et al., 2020).

### Overproduce human AQPs in *P. pastoris* at large scale

**Timing: 7 days**

This protocol is compatible for overproducing any human aquaporin in *P. pastoris* under the control of the AOX1 promoter. The culture is first fed with glycerol as the carbon source in order to generate bio-mass before switching to methanol for induction of AQP expression. We use a 3 L fermenter (see key resources table) to allow for close monitoring of growth conditions, which is advantageous due to the risk of methanol accumulation that could lead to hampering of cell growth.

***Alternatives:*** Overproduction can also be achieved in baffled shaker flasks, similarly to what is done in the small-scale expression protocol above, in which case methanol should be added to a final concentration of 0.5% (v/v) every 24 h (see EasySelect Pichia Expression Kit manual for details). However, it should be noted that while it is assumed that all methanol is consumed within 24 h in most cases, this may vary depending on growth rate. To avoid the risk of methanol accumulation, a fermenter is therefore a better alternative when available and, in our experience, leads to higher cell and protein yield.

Day 1 – Prepare the fermenter and media.

24. Streak a single colony from the desired clone on a YPD-agar plate without antibiotics and incubate it for 2 days at 30°C.
25. Inoculate 100 mL YPD-medium in a 250 mL flask with a swab of cells from the plate and grow at 30°C for 16 h. Check OD600, normally it reaches 20–25 after 16 h.
26. Prepare 1.5 L Basal Salt Medium (BSM).
27. Assemble the fermenter according to the manufacturer’s instructions (excluding any parts that should not be autoclaved).
28. Pour the BSM into the 3 L fermenter jar using one of the free ports and autoclave the fermenter vessel with BSM together with the following items:
  a. Accessory tubing e.g., glycerol tubing (excluding HCl and Ammonia tubing).
  b. 200 mL 50% (v/v) glycerol.
  c. An empty 500 mL bottle (for methanol).
  d. 20 mL 50% (v/v) Polypropylene glycol P 2,000 in H_2_O (anti-foam solution).
29. Prepare 100 mL Pichia trace metals (PTM) and sterile filter it using a 0.2 μM Corning® syringe filter.

***Note:*** The PTM-solution can be stored at 22°C for a maximum of 6 months.

Day 2 – Inoculate and feed with glycerol.

Plan to inoculate the fermenter at around 4 pm, so that the glycerol provided in the BSM will be depleted by 7–8 am on the following day.

30. Connect all tubing, equipment and bottles containing base (25% (v/v) ammonia), acid (1 M HCl) and anti-foam solution to the fermenter following manufacturer’s instructions.
31. Calibrate the pH electrode which should be pH 4 and 7 and wash the pH electrode with 70% (v/v) ethanol before inserting it into the fermenter vessel.
32. Calibrate the oxygen detector, setting the dissolved oxygen (DO) to 0% when the detector is disconnected and 100% when the detector is reconnected (gas flow 1 L/min and stirring of 500 rpm). Wash the oxygen detector probe with 70% (v/v) ethanol before inserting into the fermenter vessel.
33. Adjust the pH of the BSM to 5 by adding base.
34. Add 6.5 mL of PTM to BSM.
35. Inoculate BSM in the fermenter with the 100 mL overnight starter culture.
36. Set the fermenter parameters (we use temperature 30°C, pH 5.0–5.1, DO 25%, gas flow 1 L/min stirring 800–1,500 rpm) and run overnight.

Day 3 – Induce with methanol.

At around 7–8 am the following day, the glycerol in the culture will have been consumed, generating a spike in the DO graph. The time point could vary depending on the cell density of the starting culture and growth of the strain being used. After a supplementary glycerol feed during the day, the methanol feed is started in the afternoon which will induce the AQP expression.

***Note:*** The appropriate feeding speeds will depend on the pump and tubing. Hence, the speeds given in the protocol are merely indicative.

37. Once observing the DO spike, feed the culture with additional 200 mL of 50% (v/v) glycerol mixed with 2.4 mL PTM as follows:
  a. Start the feed at a medium speed of 10 mL/h.
  b. Increase it slowly to 27 mL/h throughout the day while carefully observing the DO which should be maintained around 20–25.
  c. Add a few drops of antifoam if it is foaming a lot.
38. At around 3 pm, switch off the glycerol feed.
39. Wait to see a spike in DO to make sure that any accumulated glycerol has been depleted.
40. Switch the feed to 99% (v/v) methanol (400 mL) mixed with 4.8 mL PTM and start feeding at a medium pump speed (10 mL/h) for 20 min.
41. Switch off the methanol feed, and wait for about 2 h for a DO spike to appear.
42. Once seeing the DO spike, switch on the methanol feed at low speed (2 mL/h) in order to avoid accumulation. Estimate a total use of 400 mL of methanol during 48 h of induction.
43. Add 500 μL anti-foam and leave it for the next 16 h. If the culture is already foaming a lot, do the following:
  a. Switch off the gas, wait for it to settle and switch it back on.
  b. Repeat this until it can run calmly with both gas and methanol again.
  c. Wait 10 min to make sure foaming doesn’t start again.

Day 4 – Continue the methanol feed.

44. Increase the methanol feed pump speed gradually to 7 mL/h throughout the day.
  a. Increase the methanol feed pump if turning off the methanol resulted in a DO spike within 30 s.
  b. Switch the methanol feed back to the same speed as before if the spike does not appear in 30 s.
45. Stabilize the DO around 25 and leave it with the same feeding rate for next 16 h.
46. Make sure there is enough methanol left in the bottle to last for 16 h.

Day 5 – Harvest cells.

47. Harvest the cells after 48 h induction (or whatever time point you found optimal in the small-scale expression screening).
  a. Turn off everything except for stirring (500 rpm) and gas flow (1 L/min).
  b. Connect the sample collecting tube and transfer the culture into centrifugation flasks.
48. Centrifuge at 9,000 × *g* for 30 min using 1 L polypropylene bottles to harvest the cells.
49. Discard the supernatant and spoon the cells into a 2–3 L plastic bag. Flatten the content and weigh the cells (we typically obtain 600 g of wet cells from a 2 L fermenter culture).
50. Disassemble and clean the fermenter according to the manufacturer’s instructions.

**Pause point:** The cells can be stored at −20°C for at least a month without affecting the yield of the protein after purification but for longer time storage, keep the cells at −80°C.

### Prepare membranes from *P. pastoris* cells expressing human AQP

**Timing: 1day**

This protocol is optimized to prepare membrane from 100 g of cells using a BSP BioSpec bead beater (see key resources table).

***Alternatives:****Pichia pastoris* cells can be broken using other cell disruption methods as well. We have previously successfully used French press (Frick et al., 2014) as well X-press systems (Nyblom et al., 2009). In terms of affordability and efficiency, we have found the bead beater to be the best option, however for proteins that are highly sensitive to heat, other methods may be more advantageous. Sonication does not generally work well for yeast due to the presence of a cell wall.

51. Pre-chill the stainless-steel breaking chamber and 200 mL glass beads at −20°C for 1–2 h or 16 h.
52. Prepare 200 mL breaking-buffer and leave it to cool down in the fridge or in an ice-bath.
53. Weigh 100 g of fresh or frozen cells in a 400 mL glass beaker.
54. Add 100 mL breaking-buffer and stir at room temperature for 30 min to thaw and resuspend the cells.
55. Add 2 mL of 100 mM PMSF, resulting in a final concentration of 2 mM PMSF in the sample.
56. Fill the ice jacket with ice and assemble the rotor on the rotor base according to the manufacturer’s instructions.
57. Run the bead beater for 24× 30 s with 30 s of pause in between each run. Add more ice to the ice jacket if necessary, as the bead beating generates heat.
58. Decant the sample carefully into a 500 mL polypropylene centrifuge bottle. Avoid including as much glass beads as possible.
59. Wash the beads by adding 100 mL breaking-buffer and add the washing solution to the sample.
60. Centrifuge the sample using a JLA-10,500 rotor at 16,500 × *g* for 40 min at 4°C to spin down cell debris and unbroken cells.
61. Transfer the supernatant to several Ti45 ultracentrifuge tubes and collect the membranes through ultracentrifugation at 200,000 × *g* for 1 h at 4°C.
62. Discard supernatant and estimate the amount of collected membranes by weighing the tubes before and after transferring the pellets to a potter homogenizer.
63. Wash the membranes by adding 10 mL urea buffer per gram of membrane and homogenize until the pellet has been completely suspended in the buffer.

***Note:*** This step removes loosely bound proteins and peripheral membrane proteins.

64. Transfer the homogenized sample to ultracentrifuge Ti45 tubes. Wash the homogenizer with an additional 3 mL of urea buffer and add this to the tubes in order to completely transfer all the sample. Centrifuge at 200,000 × *g* for 2 h at 4°C.
65. Remove the supernatant and transfer the pellet to the homogenizer. Weigh the pellet as in step 62, add 10 mL of membrane buffer with added 1 mM PMSF and 2 mM EDTA pH 9 (final concentrations) per gram of membrane and homogenize.

***Note:*** Please be aware that the pellet may be quite soft in parts so make sure to not discard too much by accident.

66. Transfer the sample to Ti45 ultracentrifuge tubes and wash the homogenizer with membrane buffer as in step 64. Centrifuge again at 200,000 × *g* for 1.5 h at 4°C.
67. Remove the supernatant, weigh the membrane pellet and homogenize in 0.5 mL/g membrane-buffer. Divide the resuspended membranes into 10 mL aliquots in 15 mL Falcon tubes and freeze.

***Note:*** We typically obtain 20 g of washed membranes from 100 g of cells.

**Pause point:** Membranes can be flash-frozen in liquid nitrogen and stored for a month at −20°C before proceeding with protein purification, or saved at −80°C for long-time storage.

***Optional:*** An additional wash step in 20 mM NaOH can be added after the urea wash to further remove unwanted proteins. Please be aware that the standard ultracentrifuge tubes may not be compatible with NaOH in which case alternative tubes must be used (see manufacture’s guidelines regarding which tubes are suitable). In our experience, the NaOH wash often does not give any additional improvement in purity of the final human AQP and we most often choose to omit it. However, this should ideally be checked for each new protein.

### Purify AQP from the prepared membranes

**Timing: 3 days**

Since finding the optimal detergent, detergent concentration and solubilization time is crucial for efficient AQP extraction from the *P. pastoris* membrane, a detergent screen should be performed. If a suitable detergent has already been identified, please proceed to day-2.

Day-1 Screen for a suitable detergent.

68. Choose a set of 5–10 detergents and make stock solutions at 2× final solubilization concentration in 1 mL freshly prepared solubilization-buffer.

***Note:*** A good starting point for the final concentration is 10× CMC. In our laboratory, our initial screen consists of six detergents: n-Dodecyl-β-D-Maltopyranoside (DDM), n-Decyl-β-D-Maltopyranoside (DM), Octyl β-D glucopyranoside (OG), Nonyl β-D-glucopyranoside (NG), Octyl Glucose Neopentyl Glycol (OGNG) and N-Tetradecyl-N-N-Dimethyldodecylamine N-oxide (LDAO) at final concentrations of 1% (w/v), 1% (w/v), 4% (w/v), 2.5% (w/v), 2% (w/v) and 1.5% (w/v) respectively.

69. Thaw the frozen membranes, if necessary, and aliquot 30 μL of membrane suspension for each detergent in a 1.5 mL Eppendorf tube.
70. Add 30 μL of the 2× detergent stock solution and incubate the tubes at 4°C with gentle shaking for a total duration of 3 h. Collect 10 μL samples after 1 and 2 h.
71. Spin down non-solubilized material in a chilled benchtop centrifuge at 15,000 × *g* and 4°C for 30 min.
72. Transfer the supernatant to a separate tube and mix it with 10 μL 2× SDS loading-buffer.
73. Add 10 μL 2× SDS loading-buffer to the remaining pellet and resuspend it.

**Pause point:** The samples can be frozen and stored at −20°C for up to a month before further analysis.

**CRITICAL:** Do not boil the samples in the SDS loading-buffer before loading on SDS-PAGE for electrophoresis. In our experience, this causes membrane proteins in general and AQPs in particular to aggregate.

74. Run a western blot (see “Small-scale screening of AQP expression”, steps 14–22).
75. Compare the intensity of the AQP bands in the supernatant and pellet for each detergent in order to estimate the solubilization efficiency, with solubilized AQP being found in the supernatant.

**Figure 2:**
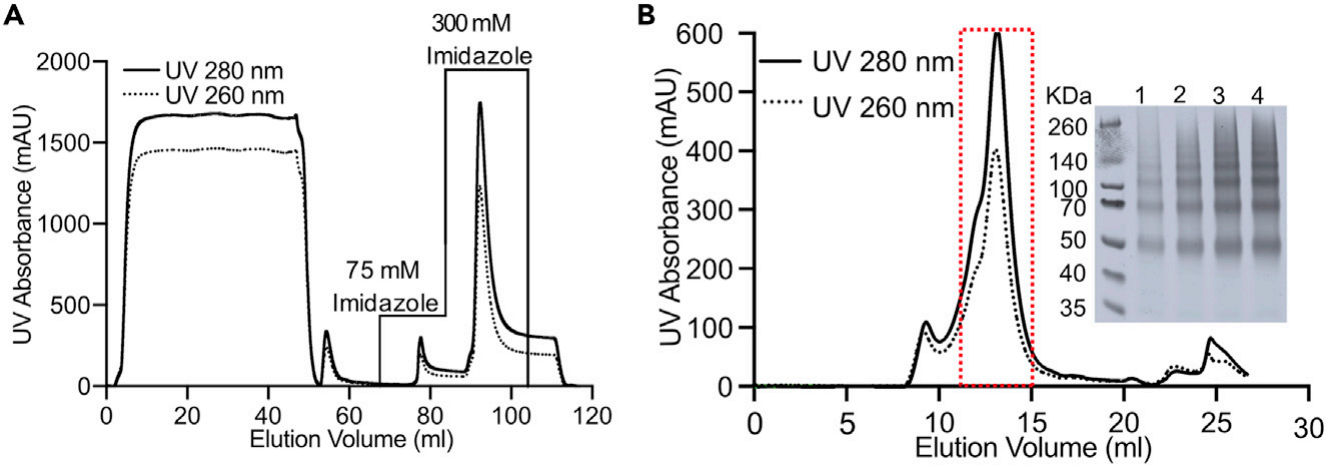
Purification of human AQPs (A) A representative IMAC chromatogram for AQP4. Following a washing step with 75 mM Imidazole, bound his-tagged AQP was eluted using 300 mM Imidazole. (B) A representative size-exclusion chromatogram for AQP4 from a Superdex 200 Increase 10/300 column using a flow rate of 0.4 mL/min. UV absorbance at 260 nm and 280 nm are shown as dotted and solid lines respectively. The insert shows the SDS-PAGE run of the main elution peak (marked with red a dotted box) showing the typical laddering pattern of AQPs that we obtain using the conditions and material that are standard for SDS-PAGE analysis our laboratory. Note that, in these conditions, we don’t see a band for the AQP4 monomer, however this is most often present for other AQPs.

***Note:*** Human AQPs produced in *P. pastoris* often appears as several bands on SDS-PAGE electrophoresis/western blot corresponding to different oligomeric state of the protein (Figure 2B).

Day-2 Solubilize membranes and run IMAC purification.

The protocol below describes the typical FPLC-based purification procedure for human AQPs used in our laboratory and comprises immobilized metal affinity chromatography (IMAC) followed by size-exclusion chromatography (SEC). The IMAC step may be replaced by an alternative affinity chromatography method or ion exchange chromatography. In the protocol we use OG as detergent but this, as well its concentration, incubation time and temperature, should be replaced with whatever was shown to be most suitable in the detergent screening step above. We recommend running all the chromatography steps at 4°C.

***Alternatives:*** For the IMAC-step, it is possible to use a gravity flow column (Nyblom et al., 2009) or a batch protocol (Karlsson et al., 2003) instead of the HisTrap protocol described here, in particular when protein yields are very high. However, we find that using an FPLC-system is superior in terms of being able to closely follow the elution and avoid any impurities.

76. Thaw 10 mL of *P. pastoris* membranes containing human AQP and transfer to a small beaker. Dropwise add 10 mL of solubilization-buffer containing 8% OG (w/v) (2× final concentration) while stirring gently. Incubate with gentle stirring for 2 h at 4°C.
77. Dilute the sample with 30 mL of solubilization-buffer and transfer it to ultracentrifuge tubes.
78. Spin down insolubilized material at 250,000 × *g* for 45 min at 4°C using a Beckman Ti70 rotor.

**CRITICAL:** After solubilization it is important to dilute the sample immediately after the incubation step in order to reduce the detergent concentration. Longer incubation at high detergent concentration can degrade the protein resulting in lower yield.

79. Collect the supernatant and add imidazole to a final concentration of 10 mM.

**CRITICAL:** Adding imidazole to a final concentration of 10 mM in the supernatant of the solubilized membranes before loading on the column for IMAC helps to reduce the non-specific binding of proteins to the Ni-NTA matrix of the HisTrap column.

80. Attach a HisTrap HP 5 mL column to an FLPC-system and equilibrate it with with 25 mL of buffer-A using a flow rate of 1 mL/min or according to the column manufacturer’s instructions.

***Alternatives:*** HisTrap columns may be run using a peristaltic pump or syringe instead.

81. Load the sample at 0.8 mL/min and continue to run buffer-A at 1 mL/min through the system until the baseline returns to zero.
82. Wash the column with 15 mL of buffer-B at 1 mL/min flow rate.
83. Elute the bound proteins with 20 mL of buffer-C at 1 mL/min and collect 1 mL fractions (Figure 2A).

***Note:*** We find that the step-wise elution protocol described above works well for the AQPs we have studied so far. However, the imidazole concentration in each step should ideally be experimentally evaluated for every new protein. This can be done by including more steps and imidazole concentrations or by running a gradient elution protocol and carefully evaluating all fractions using SDS-PAGE and, if necessary, Western blot.

84. Analyze the fractions by SDS-PAGE and pool the desired fractions.
85. Concentrate to up to 10 mg/mL using a spin concentrator (see key resources table) with a molecular weight cut off of 50 kDa.

**CRITICAL:** Although the AQP tetramer has a molecular weight of ∼120 kDa, it is our experience that using a spin filter with a molecular weight cut off of 50 kDa is helpful to reduce sample loss. In addition, reducing the centrifugation speed and careful pipetting of the sample up and down a couple of times during the concentration step also increases sample yield.

**Pausepoint:** After the addition of 10% (v/v) glycerol, the purified protein sample can be aliquoted, flash frozen in liquid nitrogen and stored for a month at −20°C or −80°C for several months before proceeding to the next step.

**CRITICAL:** For many membrane proteins, prolonged storage in high imidazole concentration leads to precipitation. In these cases, it is necessary to first remove the imidazole using a desalting column or by directly proceeding to size-exclusion chromatography without freezing the sample. For proteins that are extra sensitive, we recommend to remove the imidazole as soon possible after it has been eluted from the column, even if it is not going to be frozen. In these cases, the most convenient option is to proceed to size exclusion chromatography the same day.

Day-3 Purify the sample further using size-exclusion chromatography.

To further increase sample purity and homogeneity, a size-exclusion chromatography step (SEC) is added after the IMAC. We typically use a Superdex 200 Increase 10/300 GL column (Cytiva, US) however this can be exchanged for another size-exclusion column with an appropriate separation range. In addition to serving as a polishing purification step, SEC is an important analytical tool for assessing the presence of aggregates or multiple oligomeric states that may warrant detergent and/or buffer optimization.

86. Equilibrate the column with 1.5 column volumes of SEC-buffer.
87. Inject 500 μL of the concentrated (10 mg/mL) IMAC sample into a 1 mL loop and load the sample on the column.
88. Collect the eluate in 1 mL fractions and analyze the interesting fractions by SDS-PAGE.

***Note:*** The AQP tetramer typically elute at 12–14 mL from a Superdex 200 Increase 10/300 GL column (Figure 2B). If aggregates are present, for example due to the use of non-optimal detergent or buffer conditions, they will elute at the column void volume.

89. Pool the selected fractions together, and, if necessary, concentrate as above to the desired concentration.

**CRITICAL:** It is possible to aliquot, flash-freeze and store the purified protein after SEC. Before freezing, we recommend to add 10% (v/v) glycerol to the purified protein or, alternatively, to include 10% (v/v) glycerol in the SEC buffer. However, up to 50% of the AQP may aggregate at every freeze and thaw procedure. Therefore, it is advisable to refresh the protein after freezing through an additional SEC step before performing follow-up experiments. For proteins that are prone to aggregate during or immediately after purification, we recommend to include 10% (v/v) glycerol in all purification buffers, including IMAC.

## Expected outcomes

Using this protocol, we typically obtain 10–50 mg of >95% pure human AQP per 100 g of cells from a well-expressing clone grown in a fermenter.

## Limitations

The results from the small-scale screening of AQP expression should not be considered quantitative but is rather a relative comparison of protein expression in selected clones in order to identify the best clone for subsequent large-scale culturing. It can nevertheless be useful to include a clone that is known to express a protein at high levels in the small-scale screening experiment as a positive control. Note that it is not sufficient to include it in the western blot only, it should be grown under the same conditions as the clones of interest.

Breaking of *P. pastoris* cells using a bead beater can generate heat, which may cause high levels of wrongly folded or aggregated protein in the sample. If this is a problem, it may help to reduce the number of cycles and/or increase the pause in between them. Alternatively, other cell disruption methods could be used, see “Membrane preparation from *P. pastoris* cells expressing human AQP” above.

## Troubleshooting

### Problem 1

It can be difficult to identify a suitable *P. pastoris* clone (steps 5–23) for optimal expression of the targeted AQP.

### Potential solution

In principle, a higher copy number of the AQP gene in the *P. pastoris* genome gives a higher yield of recombinant protein, as explained in steps 1–4. If the small-scale expression does not result in the identification of a suitable clone, increase the number of clones selected from the 2,000 μg/mL zeocin plate to 15 or more (up to 50 could be needed). If the problem persists, consider changing the gene construct as the position of tags and lengths of N- and C-termini may have a substantial effect on the expression level.

### Problem 2

Accumulation of methanol in the fermenter during induction of AQP overexpression (steps 40–46) is toxic for the cells and can cause poor protein production yields.

### Potential solution

Increase the methanol feed very slowly and only when the entire added methanol is being consumed directly, as indicated by the appearance of a DO spike (step 44) when the methanol feed is switched off.

### Problem 3

The protein aggregates during or after purification, as indicated by visible precipitation in the fractions or a substantial peak at the void volume (steps 86–89).

### Potential solution

Add 10% (v/v) glycerol to all purification buffers and/or remove imidazole immediately after eluting the protein from the IMAC-column. If the problem persists, consider changing to a different detergent.

### Problem 4

The protein cannot be found in the IMAC elution fractions (step 83).

### Potential solution

Determine the appropriate imidazole concentration for the IMAC wash and elution steps by adding more steps at additional imidazole concentrations or running a gradient elution step. Analyze the resulting fractions carefully using SDS-PAGE and, if necessary, Western blot, in order to localize the protein of interest and modify the IMAC protocol accordingly.

## Resource availability

### Lead contact

Further information and requests for resources and reagents should be directed to and will be fulfilled by the lead contact, Prof. Susanna Törnroth-Horsefield Email: Susanna.Horsefield@biochemistry.lu.se.

### Materials availability

All unique/stable reagents generated in this study are available from the lead contact with a completed Materials Transfer Agreement.

## Data Availability

This study did not generate/analyze datasets and codes.

## References

[bib1] Frick A., Eriksson U.K., de Mattia F., Oberg F., Hedfalk K., Neutze R., de Grip W.J., Deen P.M., Tornroth-Horsefield S. (2014). X-ray structure of human aquaporin 2 and its implications for nephrogenic diabetes insipidus and trafficking. Proc. Natl. Acad. Sci. U S A.

[bib3] Karlsson M., Fotiadis D., Sjövall S., Johansson I., Hedfalk K., Engel A., Kjellbom P. (2003). Reconstitution of water channel function of an aquaporin overexpressed and purified from *Pichia pastoris*. FEBS Lett..

[bib4] Kitchen P., Salman M.M., Halsey A.M., Clarke-Bland C., MacDonald J.A., Ishida H., Vogel H.J., Almutiri S., Logan A., Kreida S. (2020). Targeting aquaporin-4 subcellular localization to treat central nervous system edema. Cell.

[bib6] Nyblom M., Frick A., Wang Y., Ekvall M., Hallgren K., Hedfalk K., Neutze R., Tajkhorshid E., Törnroth-Horsefield S. (2009). Structural and functional analysis of SoPIP2;1 mutants adds insight into plant aquaporin gating. J. Mol. Biol..

[bib7] Öberg F., Sjöhamn J., Conner M.T., Bill R.M., Hedfalk K. (2011). Improving recombinant eukaryotic membrane protein yields in *Pichia pastoris*: the importance of codon optimization and clone selection. Mol. Membr. Biol..

